# Turner Syndrome where are we?

**DOI:** 10.1186/s13023-024-03337-0

**Published:** 2024-08-28

**Authors:** Najma Khan, Anam Farooqui, Romana Ishrat

**Affiliations:** 1 Centre for Interdisciplinary Research in Basic Sciences, Jamia Millia Islamia University, New Delhi, 110025 India; 2 Biomedical Informatics Centre, Indian Council of Medical Research-National Institute for Research in Reproductive and Child Health, Mumbai, Maharashtra 400012 India

**Keywords:** Turner syndrome, Monosomy X, Cardiovascular malfunction, Autoimmune disorders, Metabolic imbalances, Osteoporosis, Neurocognitive deficits, Hearing loss

## Abstract

Turner syndrome (TS) results from the loss of one X chromosome in phenotypic females, leading to a range of complications such as short stature, cardiovascular issues, autoimmune disorders, metabolic imbalances, osteoporosis, neurocognitive deficits, hearing loss, abnormalities in endocrine functions, infertility, disruptions in bone metabolism, and neurocognitive deficits. These diverse clinical manifestations necessitate a comprehensive and multidisciplinary approach to diagnosis and management. Growth hormone therapy stands out as a fundamental treatment for addressing the challenges associated with TS. Ongoing clinical and genomic advancements contribute to an evolving understanding of TS, shedding light on its complexities and potential therapeutic interventions. Despite progress, further research is crucial to identify candidate pathways and critical biomarkers that can alleviate the syndrome’s burden. By uncovering these insights, we aim to empower individuals with TS, enhancing their overall functioning and quality of life. In this review, we have explored the prevalent co-morbidities associated with TS, drawing insights from the current literature.

## What is Turner Syndrome?

Turner Syndrome (TS) is a chromosomal disorder characterized by the partial or total loss of one of the two X-chromosomes in female cells due to sporadic nondisjunction. It is among the most common genetic disorders occurring globally. TS affect all geographical regions and cultures equally. It is estimated to occur in 1 in 2500 live-birth females and requires a chromosomal analysis for definitive diagnosis. Multiple karyotypes (e.g., 45, X monosomy, 45, X/46, XX mosaicism, and structurally abnormal X) have been identified that are linked to varying presentations along the TS phenotype spectrum. Girls with 45,X monosomy typically have the most severe phenotype [1]. Turner patients have significantly higher morbidity and mortality rates compared to normal individuals. [2], as an increase in the mortality rates in TS is expected by about 4- to 5-fold. The life expectancy of an individual affected with TS reduces by up to 13 years [3]. It was first described by Ullrich in Germany and as a distinct entity in 1938, by Turner, in seven females with sexual infantilism, cubitus valgus, and webbed neck, and its chromosomal basis was first recognized by Ford et al. in 1959 [4].

Turner Syndrome’s phenotype and genotype correlations are generally poor, and the exact cause remains incompletely understood. However, it is believed to result from both genomic imbalances induced by sex chromosome genes and the additive effect of epigenetic factors on linked genes within gene networks. The short stature homeobox-containing gene (SHOX) regulates growth, and its haploinsufficiency contributes to the characteristic short stature in TS. Among cases that are detected in live neonates, there is considerable heterogeneity of phenotypic features. Short stature and gonadal dysgenesis stand out as the most consistent symptoms among all those observed. [5]. Signs and symptoms vary among those affected depending upon the karyotype. Often, a short and webbed neck, low hairline at the back of the neck, low-set ears, short stature, and swollen feet and hands are seen at birth. Generally, they are without menstrual periods and do not develop breasts. Due to premature ovarian failure, they are estrogen deficient and almost all women with TS are infertile, although some of them may conceive with assisted reproduction. Other manifestations include hypertension, elevated hepatic enzymes, and middle ear infections [6] increased susceptibility to certain diseases such as hypothyroidism, renal malfunctions, osteoporosis, lymphedema, congenital heart problems, deficiencies in cognitive abilities, such as visual-spatial skills, motor function, attentional abilities and autoimmune such as type 1 and type 2 diabetes through an unknown mechanism [7]. TS girls exhibit distinct growth patterns, with a sharp decline in growth rate until 2 years of age, followed by gradual increase until puberty. After puberty, the growth rate increases slightly due to the lack of epiphyseal closure [8].

## Genetic aspects of Turner Syndrome

**Fig. 1:**
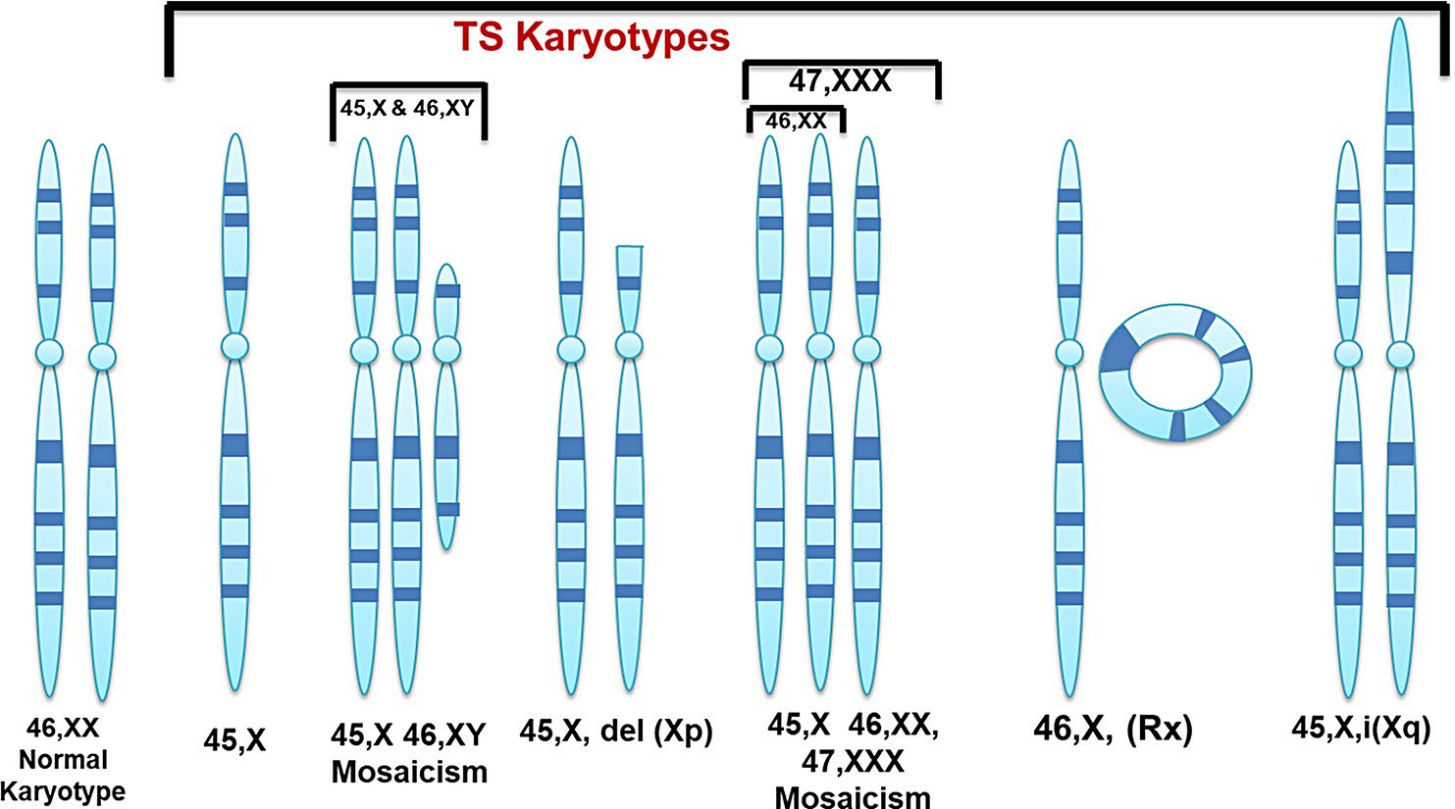
Normal Karyotype and Karyotypes found in TS Patients like mosaicism of X chromosome, deletion of Xp or Xq, ring X (46,X, rX), and isochromosome Xq

Aside from monosomy X (45,X), which is the most prevalent in TS, other cases have been described with mosaicism, in which the 45, X cell line is accompanied by one or more additional cell lines with a complete or structurally abnormal sex chromosome (X or Y) (Fig. 1). Structural abnormalities of the sex chromosome (X) can be caused by deletions of the short or long arms (Xp-, Xq-, respectively), creation of the ring (rX) (Fig. 1), or duplication of the long arm to produce an isochromosome (isoXq) (Fig. 1). Some mosaic females with the Y chromosome carry more cell lines (45, X/46, XX; 45, X/46, XY) (Fig. 1). Mosaicism is estimated to be relatively common in affected individuals (67–90%) [9]. Approximately 99% of all fetuses with TS result in spontaneous termination during the first trimester and as many as 15% of all spontaneous abortions have the 45, X karyotype [10]. TS is primarily not inherited, with most cases involving monosomy involving the mother’s X chromosome. It is a sporadic event, with no increased risk of recurrence in subsequent pregnancies. However, if a balanced translocation occurs or if the mother has 45, X mosaicism is restricted to her germ cells. In rare cases, partial deletion of the X chromosome can result in TS inheritance by the next generation [5].

## Turner Syndrome and comorbidities

**Fig. 2:**
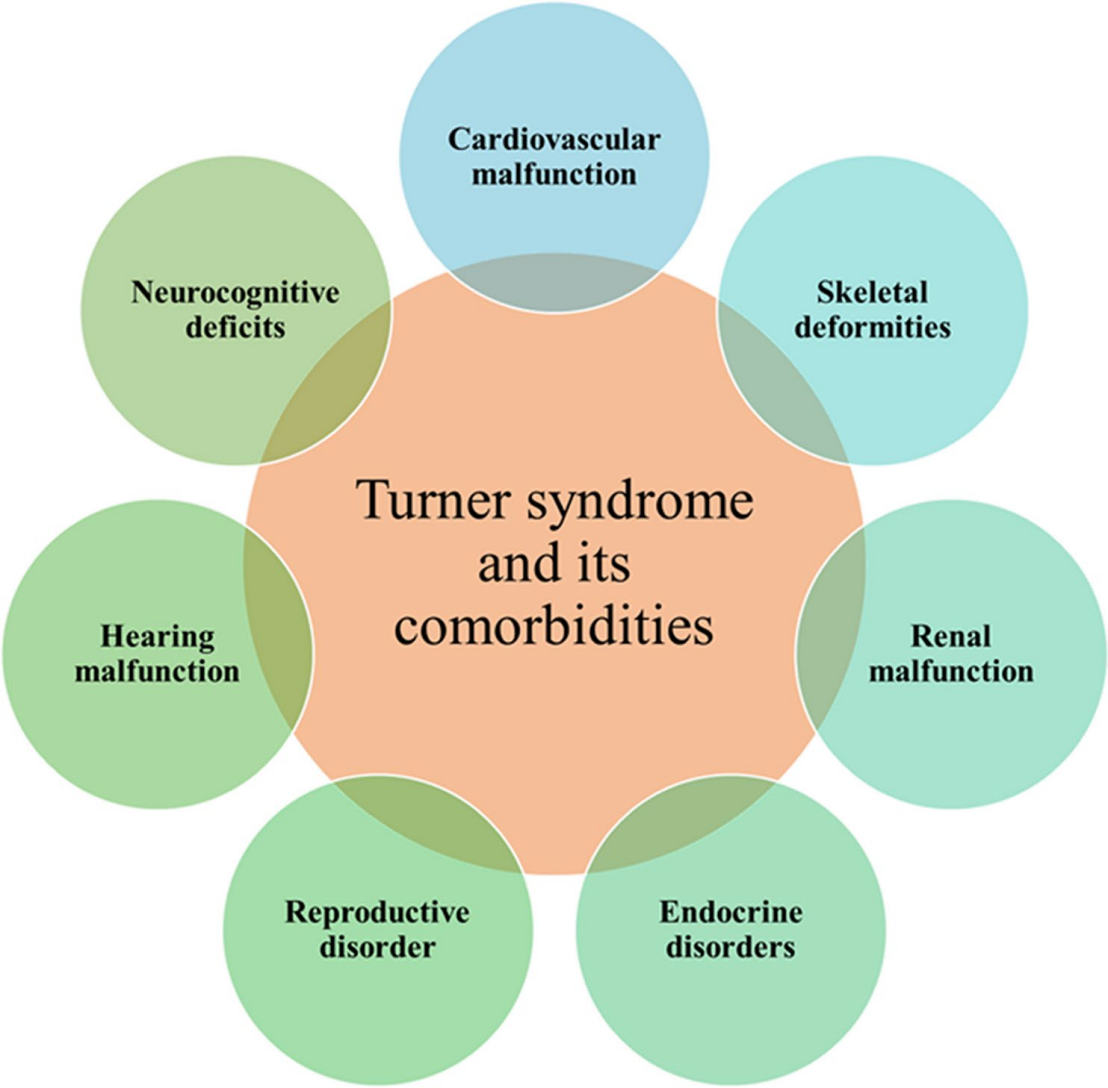
TS and its comorbidities: - Cardiovascular malfunction, Skeletal deformities, Renal malfunction, Endocrine disorders, Reproductive disorders, Hearing malfunction, neurocognitive deficits

TS is characterized by various comorbidities that can impact different systems within the body. Among these comorbidities are cardiovascular issues, autoimmune disorders, metabolic imbalances, osteoporosis, neurocognitive deficits, hearing loss, abnormalities in endocrine functions, infertility, and disruptions in bone metabolism, etc. (Fig. 2). These diverse challenges underscore the multifaceted nature of TS, necessitating comprehensive medical care and management strategies to address the wide range of potential health concerns. In this review, we have explored the prevalent co-morbidities associated with TS, drawing insights from the current literature.

### Cardiovascular malfunction and Turner Syndrome

TS has been associated to a substantial rise in the occurrence of cardiovascular malformations.

 [11], as well as high cardiovascular morbidity and fatality rates in TS patients. Although the frequency appears to rise with age, aberrant aortic dimensions are evident from childhood. Congenital heart abnormalities impact 23–50% of people with TS being one of the main causes of premature death. The mortality rate is higher in individuals with 45, X karyotypes compared to those with X mosaicism or other X structural abnormalities [12]. According to an epidemiological study, people with TS had a three-fold greater mortality rate than the general population. Notably, cardiovascular events pose a significant risk, with such incidents occurring in 41% of TS patients [13]. The most prevalent cardiovascular complications in TS are congenital heart abnormalities, aortic dissection, hypertension, and aortic dilatation. Aortic stiffness increases in TS females before the beginning of puberty [14]. Congenital cardiac defects, like coarctation of the aorta and bicuspid aortic valve, are found in approximately one-third of TS patients, with bicuspid aortic valve being the most prevalent, occurring in approximately 25% of TS patients [15], the link between TS and the aortic bicuspid valve is most likely due to several complex mechanisms, one of which appears to be the action of TIMP1 and TIMP3. The imbalance of TIMP1 and TIMP3 contributes to the increased vulnerability to aortic morphological abnormalities. The increased prevalence of bicuspid aortic valve in TS is also linked with karyotype and is most common in women with the 45, X karyotype. Other left-sided congenital lesions, like abnormal pulmonary vein drainage, coarctation of the aorta, mitral valve dysplasia, subaortic obstruction, and coronary anomalies, are frequently observed in TS individuals. These cardiovascular complexities contribute significantly to the shortened life expectancy of TS individuals. Hypertension, being a significant risk factor for cardiovascular complications, affects approximately 50% of TS patients [16]. TS individuals face a heightened risk of mortality primarily due to aortic dissection aneurysms. In comparison to non-TS individuals, young TS individuals exhibit a significantly smaller aortic diameter. To mitigate the risk of aortic dissection, especially in those aged 18 and above, aortic surgery becomes a recommended intervention. Specifically, individuals with TS are advised to undergo aortic surgery when their ascending aortic size index exceeds 2.5 cm/m², aiming to prevent the occurrence of aortic dissection and associated complications [13]. Cardiovascular disorders in TS include early-onset hypertension, ischemia, and stroke, with hypertension often attributed to coarctation of the aorta, kidney dysfunction, obesity, and metabolic syndromes. In conclusion, understanding the diverse cardiovascular manifestations in TS is crucial for early diagnosis and intervention, given the elevated risk of life-threatening complications. More research on structural, genetic, and epigenetic aspects is necessary to obtain an important understanding of the complex interactions between TS and cardiovascular diseases.

### Skeletal malformations and Turner Syndrome

TS is linked with skeletal abnormalities, like short stature, angular deformities in the lower and upper limbs, spine deformities in both coronal and sagittal planes, changes in bone growth, and the early onset of osteoporosis. It was previously believed that the gonadal dysgenesis observed in TS patients was linked to these skeletal abnormalities. But recent studies on genetic variations in TS patients have identified a deficiency of the gene encoding the short stature homeobox (SHOX), which is a bone regulating center for longitudinal growth. With its highest expression in the mesomelic area of the limbs, the SHOX gene (Table 1) has significant expression in the perichondrial layer along the diaphysis of long bones. The gene’s (SHOX) dosage insufficiency can cause bowing and shortening in the forearms and lower legs, potentially leading to short stature, Mesomelic Growth, Delayed Skeletal Maturation, Upper Extremity, Spinal Deformity, Lower Extremity, Genu Valgum, Brachymetatarsia, Coalitions, and cubitus varus. Short stature and delayed skeletal development are probably caused by the interplay between SHOX haploinsufficiency and estrogen deprivation. Additionally, the time of skeletal development and growth plate fusion is believed to be influenced by the SHOX gene [17]. TS Women’s have approximately 25% more chances of bone fractures as compared to normal individuals [18] with the relative risk ranging from 1.16 to 2.16 [19]. These fractures occur in both trabecular and cortical bones, generally affecting the femoral neck, metacarpal bones, lower spine, and forearm [18]. Skeletal fragility in TS is most likely induced by a set of factors. These factors include inherited skeletal dysplasia or defects linked to the underlying chromosomal abnormality, reduced bone mineral density (BMD) and the onset of osteoporosis over time, a vitamin D deficiency, a higher risk of falls as a result of visuospatial cognitive dysfunction, and hearing or vision loss. Additionally, impaired balance is often observed in TS individuals [19]. The increased risk is noticeable during childhood, especially for wrist fractures, and after the age of 45, due to osteoporosis. Due to increased fracture risk, women with TS have low bone mineral content in cortical bone and the microarchitecture of their trabecular bone is weakened. Bone metabolism is a complicated interplay of physical activity, genetics, food, local growth factors, and hormones. Additionally, young TS girls already have reduced estradiol production [18].

Other TS-related co-morbidities, such as abnormal liver or thyroid function, inflammatory bowel disease, and celiac disease may potentially contribute to skeletal fragility [19]. TS patients are also more likely to develop scoliosis and kyphosis, while upper and lower extremity anomalies may be identified during clinical examination, surgical procedures are rarely required unless symptomatic [17].

Low BMD is common in adult TS women, with estrogen insufficiency being a significant modifiable risk factor. Estrogen is the primary hormonal regulator of bone health, playing a significant role in bone mass accumulation throughout skeletal growth, skeletal balance in adulthood, and accelerated bone loss during menopause. Girls with spontaneous menstruation maintain normal BMD, while those with absent menarche experience decreased BMD. Hormone replacement therapy positively impacts bone health and prevents osteoporosis by maintaining bone mineral density and promoting long-term bone health. Healthy lifestyle choices, weight-bearing activities, consuming enough calcium and vitamin D through diet, routinely checking for vitamin D deficiency, and continuing hormone replacement therapy until the natural age of menopause are all preventive strategies to maximize bone health [19].

### Renal malfunctions and Turner Syndrome

Renal malformations are common in TS individuals, with approximately one-third of them having structural abnormalities in the renal system [20]. The most prevalent renal abnormality found in TS patients is horseshoe kidney, while other anatomical defects include crossed ectopia and duplex collecting systems. These malformations can lead to an increased risk of urinary tract infections, hypertension, recurrent hematuria, and chronic kidney disease [21].

Ultrasonography and a DMSA (dimercaptosuccinic acid) test are recommended to detect renal malformations early in individuals with TS. These imaging techniques aid in the identification of any malformations as soon as possible, enabling the commencement of appropriate therapy and the avoidance of further complications [22].

Numerous case studies have been carried out to assess the frequency and nature of renal malformations in TS individuals. A study evaluating 141 TS patients found that 33% of them had renal malformations [20]. Another study analyzing 82 Turkish children with TS reported that 37.8% of the patients had different renal malformations [23]. In addition, a case study reported a 17-year-old female with mosaic ring TS and horseshoe kidney [24]. Another case study reported a five-year-old girl with a horseshoe kidney and growth retardation as the only symptom of TS [25]. These findings highlight the need of early detection and adequate treatment of renal problems in individuals with TS. Renal abnormalities are quite prevalent in TS patients; about 30% of affected persons have renal complications. Fortunately, there are usually no significant health risks linked to these complications. Regular health checks, including ultrasonography and DMSA tests, are essential for the early detection of renal malformations and the initiation of appropriate therapy. By addressing these issues proactively, it is possible to mitigate the risk of complications and improve the overall health and well-being of girls and women with TS.

### Thyroid malfunctions and Turner Syndrome

TS Females are at an elevated risk of developing autoimmune disorders, with autoimmune thyroid dysfunction being the most prevalent among various conditions. The high frequency of autoimmune disorders, particularly autoimmune thyroid dysfunction, in females with TS, has been a subject of significant research and clinical interest. Hashimoto’s disease, a common form of autoimmune hypothyroidism, is found in 30–50% of females with TS [17], with its prevalence rising as individuals age. By the age of 50, approximately half of the females with TS will experience clinical or subclinical hypothyroidism. Thyroid peroxidase (TPO) antibodies, a key marker for autoimmune thyroid disease, are highly prevalent in 40–45% of women with TS [3]. The actual etiology of the higher occurrence of autoimmune thyroid diseases in females with TS remains unknown; however, a number of hypotheses have been put forward to understand the phenomenon. One hypothesis is linked to ovarian dysfunction and the lack of androgen and/or estrogen synthesis. According to research, ovarian dysgenesis, a common characteristic of TS, may promote thyroid autoimmunity [26]. Another hypothesis suggests that the missing second X chromosome in TS females may lead to increased autoimmunity, which is known as the haploinsufficiency hypothesis [27]. Furthermore, it has been found that the isochromosome Xq, a common genetic rearrangement in TS, has been related to an elevated risk of autoimmune disorders, especially thyroid diseases [28].Also, a study revealed that autoantibodies (against adrenal cortex, transglutaminase, intrinsic factor, glutamic acid decarboxylase 65, and gliadin) were present in 58% of TS patients. [29]. Clinical findings have supported the complex nature of the pathophysiology of thyroid dysfunction in TS females. TS is linked to increased prevalence of thyroid diseases such as Hashimoto’s thyroiditis and Graves’ disease, with up to 30% of TS females developing thyroid diseases, primarily Hashimoto thyroiditis [30]. This indicates that TS females often experience compensated hypothyroidism before progressing to overt hypothyroidism, emphasizing the importance of regular monitoring of thyroid function in this population [31]. The high risk of autoimmunity in TS females has been recognized as one of the more prominent characteristics of the condition [26]. These findings emphasize the complex interaction between hormonal and genetic factors in the development of thyroid dysfunction in TS patients. However, the specific processes behind this increased vulnerability to autoimmunity remain poorly understood.

### Diabetes and Turner Syndrome

Metabolic syndrome encompasses various factors such as visceral adiposity, irregularities in glucose metabolism, dyslipidemia and hypertension, all of which are found in individuals with TS [32]. TS individuals are more likely to be overweight during childhood, which increases their risk of developing diabetes in adulthood. Weight increase occurs before puberty onset and does not differ between TS girls with induced and spontaneous puberty [33]. Ann Forbes and Eric Engel first reported in 1963 that TS patients had a higher incidence of diabetic mellitus [34] having incidence rate of (SIR 4.1 [95% confidence interval (CI) 2.5–6.3]) (*n* = 18) [26] and epidemiological studies indicate a relative risk of 8.2 to 11.6 (95% confidence interval (CI) 5.3–21.9) for developing type 1 diabetes and 4.38 (confidence interval (CI) 2.40–7.72) for developing type 2 diabetes (T2DM), as well as three times higher mortality rates than the general population [35]. Additionally, TS is linked to a 25% reduction in physical fitness compared to unaffected individuals [32]. However, these study’s findings are based on registries and there’s a risk of misclassification in coding. Few clinical trials have been conducted on females with TS and diabetes, and the pathophysiology of this increased risk is not fully understood, potentially involving complex genes or genetic backgrounds [17]. Impaired glucose tolerance (IGT) affects approximately 25–78% of adult TS populations, contributing to about 25% of TS related deaths [36]. Impaired glucose metabolism severity varies by karyotype, with mosaic TS patients showing normal glucose tolerance compared to monosomy X patients [17]. The prevalence of impaired glucose metabolism in TS patients appears to be higher than in healthy controls and women with primary ovarian failure. All TS women, including young girls who are not obese and have appropriate insulin sensitivity, exhibit this difference [37]. A study found that T2DM rates were 9% for TS patients having X chromosome with a deleted long arm (delXq), 18% for those with Monosomy X (45, X) 23% for those with an X chromosome with a deleted short arm (delXp) (Fig. 1), and an astounding 43% for those with two long arms of X chromosome (isochromosome Xq). Individuals with TS are more susceptible to Type 2 Diabetes Mellitus (T2DM) due to haploinsufficiency in genes on Xp, while having an extra copy of Xq increases the risk [38]. Patients with monosomy X have a higher risk of glucose metabolism problems, including insulin resistance and type 2 diabetes. This suggests that haploinsufficiency of genes on the Xp chromosome negatively impacts β-cell and pancreatic islet function [33]. While the specific etiology of lower glucose tolerance in TS is speculative, several hypotheses have been proposed and researched over the years. Early studies revealed that TS patients had lower insulin sensitivity than age-matched controls. But when the same theory was tested again with age- and BMI-matched controls, the insulin sensitivity in people with TS was similar to that of controls. Glucose metabolic impairments appear to exist upon glucose stimulation, and it has been observed that first-phase insulin production in response to both OGTT and IVGTT is insufficient in TS. Insulin secretion in TS is comparable to controls but insufficient to respond to glucose load, resulting in a lower insulin-to-glucose ratio. IGT may result from a delayed inhibition of hepatic gluconeogenesis caused by inadequate first-phase insulin response. Additionally, in TS, the first-phase insulin response and therefore beta-cell function tend to decline dramatically with age, indicating that IGT eventually develops into overt T2DM [17]. Despite the divergent outcomes in TS, treatment with estrogen replacement appears to be necessary for maintaining glucose homeostasis. Estrogen therapy is linked to decreased fasting insulin and glucose levels, increased fat-free mass, and improved physical fitness in patients of Type 2 Diabetes [28]. The reduced glucose tolerance is positively influenced by all factors. However, more studies are required to assess the efficacy of exogenous estrogen treatment. GH treatment has no deleterious effect on glucose levels in TS females, and HbA1c remains stable or even lowers throughout GH therapy. Insulin levels rise during GH administration, showing relative insulin resistance, but drop after treatment is stopped. Interestingly, given the high chances of cardiac disease in TS, myocardial glucose uptake in these women is reduced; nevertheless, GH treatment does not affect this. Additional research is required to ascertain the effectiveness of treating with exogenous estrogen [17].

### Reproduction and Turner Syndrome

TS patients generally exhibit primary amenorrhea or delayed puberty due to premature ovarian failure [39]. Premature ovarian insufficiency (POI) is a notable issue for TS patients and their parents. Most girls with TS experience premature ovarian failure, despite spontaneous puberty occurring in up to 38% of cases. [40]. Spontaneous pregnancy is observed in 2–5% of TS patients with a mosaic karyotype. The POI in TS is due to increased follicular apoptosis, which results in reduced fertility potential. TS females are often present with signs of estrogen deficiency due to POI [41]. Although 5–20% of the TS population experiences spontaneous menarche and regular menstruations, most of this is followed by early secondary ovarian insufficiency [42]. Ovarian dysfunction is a significant issue in girls and women with TS, leading to delayed puberty and infertility [43]. TS can also have structural and functional abnormalities in the uterus, impacting the implantation and growth of fertilized eggs. Only a quarter of individuals with TS possess a fully developed uterus in size and shape, with the majority having a slightly smaller or immature form. The average uterine volume of women with TS tends to be smaller than those with a normal karyotype, although the difference in uterine size is not significant. A range of factors, including age, duration of estrogen use, hormone replacement therapy (HRT), and the type of estrogen medication, can influence uterine size in individuals with TS. However, timely and appropriate treatment can lead to normal uterine development in TS individuals [44]. Pregnancy is scarce in TS patients and exhibit a high risk of miscarriage, stillbirth, and birth defects [45]. Only 2–5% of TS patients become pregnant spontaneously, and approximately 3.8% of TS patients have one or more live-born children. Pregnancies in TS patients, whether natural or medically aided, are at an increased risk of negative maternal and fetal outcomes compared to healthy women [46]. For instance, 23–50% of TS patients have congenital cardiac defects, and pregnancy causes a 50% increase in cardiac output, making patients with TS susceptible to aortic dissection or rupture [47]. As a result, the risk of death during pregnancy for TS patients can reach up to 2%. The colony-stimulating factor 2 receptor alpha (CSF2RA) gene present in the (PAR1) pseudoautosomal region 1 of the X chromosome is possibly involved in intrauterine lethality in fetuses with 45,X TS [10]. USP9X (located in Xp11.4) is a candidate for the failure of gonadal and oocyte development in TS. Ovarian failure in TS is associated with genes ZFX (located on Xp21.3) and USP9X (located on Xp11.4). In addition, patients who have lost the USP9X (Table 1) region experience primary amenorrhea [48]. Most TS women depend on assisted reproductive technology (ART) or alternative options such as fostering and adoption to become parents [49]. TS is a multi-system disorder that has a wide range of effects on reproductive health. Early diagnosis and specialist referral are vital for fertility preservation and HRT implementation. Spontaneous pregnancy, occurring mainly in females with mosaic TS, is uncommon and they require fertility treatments. Pregnancy is a high risk in TS females; therefore, pre-pregnancy screening, counseling, and co‐morbidity optimization are imperative. A multidisciplinary specialist team is recommended for managing pregnant women with TS to ensure optimal outcomes for both the mother and infant [50].

### Hearing malfunctions and Turner Syndrome

Hearing malfunctions are prevalent in TS individuals, leading to potential negative impacts on health and overall quality of life [51]. Hearing loss and ear disease are two of the most significant and challenging chronic health issues faced by individuals with TS. [52]. TS women frequently experience a greater rate of hearing deterioration compared to the normal individuals [53]. There hasn’t been much research done on the precise cause of TS-induced Hearing Loss, potential causes include the effect of estrogen [54], and gene and chromosome abnormalities [55]. TS individuals have an increased risk of experiencing ear and hearing issues, especially those with the 45,X and 46,X, i (Xq karyotype). [53]. Moreover, a few genes located on the short arm of the X chromosome could regulate hearing functions. The loss of these genes may cause aberrant craniofacial development and abnormalities in the anatomy of the auricles [51]. Screening for otological problems in TS involves neonatal screening, early audiometry, and regular evaluations. High-frequency audiometric testing is crucial for detecting hearing loss before it affects verbal communication. TS is associated with a high prevalence of otitis media and hearing difficulties, impacting both the middle and inner ear. The frequency of conductive hearing loss (CHL) in TS is considered to be as high as 80%, primarily due to recurrent Eustachian tube dysfunction, otitis media, and anatomical abnormalities in the external ear and skull base. The etiology of CHL in TS includes hypotonia of the tensor veli palatine muscle, abnormal tympanic ostium, lymphatic hypoplasia, and craniofacial abnormalities. Chromosomal deletions, particularly in the X chromosome’s short arm, believed to be associated with CHL, and decreased levels of (IGF-1) insulin-like growth factor 1 are associated with otitis media. Chronic, severe otitis media is also common in TS and is believed to result from obstructed drainage, possibly due to lymphatic insufficiency or skeletal dysplasia. Otitis can lead to conductive hearing loss in children, which normally resolves as the otitis decreases in the young adult stage [56]. Sensorineural hearing loss (SNHL), another hearing problem often progresses gradually from late childhood to early adulthood. The prevalence of this condition varies, with some studies suggesting that up to 90% of people are affected. Mixed hearing loss (MHL) in TS includes middle ear problems and SNHL. The challenges associated with hearing problems in TS extend beyond the physical implications, influencing social interactions, educational performance, and overall communication abilities [52]. Therefore, regular and comprehensive screening methods, including high-frequency audiometric testing, are essential in the management of otological disorders in TS patients.

### Social and psychological aspects of Turner Syndrome

Along with physical health difficulties, numerous studies have identified impairments in various psychosocial domains and overall quality of life among TS individuals. Persistent challenges in social interactions are observed throughout their lives, particularly in establishing and maintaining friendships and relationships. Parents often perceive their daughters with TS as less socially competent, with fewer friends, and spending less time socializing at an early age. Furthermore, TS females exhibit a lower likelihood of having a partner and getting married compared to their non-TS counterparts [57].

TS patients are at a higher risk of developing autism spectrum disorders, psychotic disorders, depression, anxiety, mood disorders, hyperactivity disorder, attention deficit, and schizophrenia. Young TS females often face social difficulties and challenges in adjusting to puberty [58]. They could encounter lower self-esteem and social difficulties, which can affect their overall well-being and psychopathology [59]. TS patients can also exhibit cognitive and psychosocial deficits, such as impaired memory and attention, and social interaction difficulties. They are also at an increased risk of receiving a diagnosis of neurodevelopmental including intellectual disability, and eating disorders [60]. Women with TS may exhibit deficits across various psychosocial issues such as anxiety, depression, ADHD, autism, self-esteem, and social participation. These impairments can have a detrimental impact on their overall quality of life [57]. Healthcare professionals need to be aware of these psychological and social challenges faced by TS patients and provide appropriate support and resources to aid them deal with these issues. Support groups, like the Turner Syndrome Society of the United States(TSSUS) [61] and the Turner Syndrome Foundation [62], can offer resources, education, and emotional support to patients and their families [63].

**Table 1:** Risk gene associated with TS Comorbidities

TS Comorbidities	Risk Genes
Cardiovascular malfunction	TIMP1 and TIMP3 [64]
Skeletal malformations	SHOX [65]
Renal malfunctions	CLTRN [48]
Auto immune Disorders • Thyroid malfunctions • Diabetes	IL3RA [18]
Fertility Problems	CSF2RA [10], USP9X [48], ZFX [66] KDM6A [18]
Hearing malfunctions	KDM6A [67]
Neurocognitive defects	BDNF [68]

## Management of Turner Syndrome & future aspects

TS is a multifaceted condition that affects multiple organ systems in females, necessitating a multidisciplinary approach to patient care. The present treatment focuses on growth-promoting therapy, induction and maintenance of secondary sexual characteristics, and osteoporosis prevention. Growth hormone (GH) therapy is fundamental in maintaining body stature. GH treatment accelerates growth and increases adult height, while estrogen replacement treatment induces puberty [69].TS management requires a holistic approach that includes hormonal, cardiovascular, psychosocial, and reproductive aspects. This method maximizes the health outcomes and enhances the quality of life for individuals with TS. Evidence-based practices, collaboration among medical disciplines, and patient-centered interventions are essential. Continuous research and refinement of therapeutic interventions are crucial for advancing TS management. Expanding our understanding and developing targeted treatments can improve care and outcomes for TS patients in the future.

Despite advancements in understanding TS, fundamental questions and challenges persist in areas like diagnosis, hormone replacement therapy, co-morbidities, fertility, and adult clinic establishment. Advancements in genetic and genomic factors are expected to improve patient care in TS. Next-generation sequencing, such as whole exome sequencing (WES), can uncover the genetic landscape of TS, identifying potential candidate genes that may be responsible for various phenotypic manifestations. This comprehensive approach provides a detailed genetic profile, elucidating the specific genomic alterations underlying TS. Integrating data from genomic studies with functional analyses can unravel protein regulatory networks and disease-associated pathways affected by these genomic variants. Experimental validation techniques like allele-specific PCR and Sanger sequencing can be used for validating candidate genes identified through genomic analyses, confirming their relevance to TS and their potential role in disease progression and severity. This holistic understanding of TS pathogenesis will pave the way for targeted therapeutic interventions and this type of integration holds promise for personalized medicine-enhanced therapeutic strategies and a deeper understanding of the syndrome’s genetic basis and lessens the burden of TS patients.

## Data Availability

Not applicable.
